# Unemployment Insurance: Minister's Decision

**Published:** 1921-03-26

**Authors:** W. R. L. Blakiston

**Affiliations:** Ministry of Labour, Employment and Insurance Department, Queen Anne's Chambers, 28 Broadway, Westminster, S.W. 1


### THE EDITOR'S LETTER-BOX.

**Correspondence on all subjects is invited, but we cannot in any way be responsible for the opinions expressed by our correspondents, who must give their name and address as a guarantee of good faith, but not necessarily for publication. Correspondents are reminded that brevity of style and conciseness of statement greatly facilitate early insertion.**

### Unemployment Insurance: Minister's Decision.

*To the Editor of* The Hospital.

Sir,—I am directed by the Minister of Labour to refer to the public hearing held on January 5 upon questions submitted to the Minister for determination under Section 10 of the Unemployment Insurance Act, 1920, whether the employment by a hospital supported out of voluntary contributions of a sister, a nurse, and a probationary nurse is employment within the meaning of the Act.

I am now to inform you that decisions have been given by the Minister to the following effect :—

(*a*) That the employment of a person at a voluntar hospital as a probationary nurse is such as to make that person an employed person within the meaning of the Act.

(*b*) That the employment of a person at a voluntary hospital as a nurse to nurse patients is such as to make that person an employed person within the meaning of the Act.

I am to add that the application submitted with reference to the employment of a sister at a voluntary hospital is still under consideration, and a further communication will be addressed to you on this question as soon as the Minister's decision has been given.

Your obedient Servant,
W. R. L. Blakiston.

Ministry of Labour,
Employment and Insurance Department,
Queen Anne's Chambers,
28 Broadway, Westminster, S.W. 1.
March 21, 1921.

